# Transcriptome Analysis of Long Noncoding RNAs and mRNAs in Granulosa Cells of Jinghai Yellow Chickens Illuminated With Red Light

**DOI:** 10.3389/fgene.2021.563623

**Published:** 2021-02-09

**Authors:** Ying Wang, Huiqiang Shi, Genxi Zhang, Pengfei Wu, Lan Chen, Manman Shen, Tingting Li, Xiaoyang Lv, Yunfei Gu, Jinyu Wang

**Affiliations:** ^1^College of Animal Science and Technology, Yangzhou University, Yangzhou, China; ^2^Jiangsu Jinghai Poultry Industry Group Co. Ltd, Nantong, China

**Keywords:** transcriptomics, jinghai yellow chicken, lncRNA, red light, egg production, follicular development

## Abstract

Jinghai Yellow chickens are a new indigenous breed with a dual purpose in China, but their egg laying performance is limited. Compared with white light (WL), exposure to red light (RL) can improve the egg laying performance of hens. Herein, to elucidate the molecular mechanism by which RL affects the egg laying performance, RNA sequencing was used to analyze long noncoding RNAs (lncRNAs) and mRNAs from granulosa cells of small yellow follicles from Jinghai Yellow chickens in RL and WL groups. A total of 12,466 lncRNAs were identified among the assembled transcripts, of which 168 lncRNAs were significantly different between the RL and WL groups (101 downregulated and 67 upregulated). Additionally, 1182 differentially expressed mRNAs were identified (958 downregulated and 224 upregulated). Integrated network analysis demonstrated that numerous differential mRNAs were involved in follicular development through steroid hormone synthesis, oocyte meiosis, and the PI3K-Akt signaling pathway. The impact of lncRNAs on cis and trans target mRNAs indicates that some lncRNAs play important roles in follicular development of small yellow follicles. The results provide a starting point for studies aimed at understanding the molecular mechanisms by which monochromatic light affects follicular development and egg production in hens.

## Introduction

Artificial illumination is widely used to promote reproductive performance in birds. Casey et al. (1969) concluded that shorter wavelengths stimulated reproductive development of pullets, but short wavelength illumination has a minimal effect on the rate of egg production in laying hens and breeder turkeys (Schumaier et al., 1968; Wells, 1971; Jones et al., 1982). Red light (RL) increases egg production in brown egg laying hens (Foss and White, 1983), turkeys (Pyrzak and Siopes, 1986), quails (Woodard et al., 1969), and pigeons (Wang et al., 2018). Furthermore, Pyrzak et al. (1984) suggested that hens produce fewer but larger eggs than earlier maturing hens exposed to red and white light illumination. However, Gongruttananun (2011) indicated that RL has no effect on egg production in hens, and Li et al. (2014) concluded that the wavelength of light does not affect egg production in hens. Thus, research on the effects in poultry is complicated, and few studies have focused on broiler chickens.

Five or six hierarchical follicles simultaneously reside in the ovary to support daily ovulation in hens. One single follicle from a cohort of 8–13 small yellow follicles (SYFs) 6–8 mm in diameter is selected each day to enter the preovulatory hierarchy following ovulation of the largest follicle (Johnson and Woods, 2009; Onagbesan et al., 2009). Follicles consist of an outer layer of theca inerna cells that encircle inner layers of granulosa cells (GCs) (Hsueh et al., 1984). Follicular selection is associated with GCs (Hernandez and Bahr, 2003), during which GCs become capable of producing progesterone, resulting in a surge in luteinizing hormone (Robinson and Etches, 1986). Onagbesan et al. (2009) concluded that although the oocyte is the major source of epidermal growth factor-like peptides, the granulosa and theca are the sites of action of the ligands. Elucidating the molecular mechanisms mediating GC development in hens under monochromatic light could help to improve the reproductive performance of broilers.

Long noncoding RNAs (lncRNAs) are non-translated RNAs longer than 200 bp that play an important role in pre- and post-translational mRNA processing (Cheetham et al., 2013). Increasing evidence indicates that lncRNA-mediated gene expression is critical in reproduction in both male and female animals (Liu et al., 2018; La et al., 2020). Peng et al. (2019) identified 160 mRNAs and 550 lncRNAs that differ in follicles between two different chicken breeds, many involved in oocyte meiosis, progesterone-mediated oocyte maturation, and cell cycle pathways. Meanwhile, Ren et al. (2017) revealed that 52 lncRNAs were closely correlated with divergent reproductive mRNAs in the different phases of duck ovaries. The functions of lncRNAs are closely related to the development of follicles, but data associated with the functions of lncRNAs in follicle development in chicken under monochromatic light remains limited.

In the current study, Illumina sequencing technology was employed to identify lncRNAs and mRNAs in the GCs of SYFs under red light that are related to follicle development. The results could prove useful for exploring the molecular mechanisms mediating the development of GCs under monochromatic light, and help to improve the egg laying performance of broilers.

## Materials and Methods

### Chicken Rearing and Sample Preparation

Jinghai Yellow chickens were raised by Jiangsu Jinghai Poultry Industry Group Co., Ltd. (Nantong, Jiangsu, China). After transfer to the laying house, hens were caged individually, and 300 hens were divided into RL and WL groups, with five subgroups in each. All hens were provided with water *ad libitum* and restricted food. Experimental birds were exposed to red light (RL, 660 nm) while control birds were exposed to white light (WL, 400 to 760 nm) using light-emitting diodes (Shenzhen Hongda Technology Co., Ltd, Shenzhen, China) for 16 h each day (16 h light, 8 h dark). The light intensity was 15.0 lux as measured with a TES-1336A light meter (TES Electrical Electronic Crop., Taipei, China). Egg production and egg weight were measured, and based on the pedigree record, six hens at 300 days of age with an average body weigh were selected. All efforts were made to minimize distress. SYFs with a diameter of 6–8 mm were washed carefully in cold phosphate-buffered saline (PBS; Gibco, USA) and collected using the method for collecting GCs reported previously by Gilbert et al. (1977), flash-frozen in liquid nitrogen, and stored at −80°C.

### RNA Sequencing (RNA-Seq) Sample Preparation and Sequencing

Total RNA from each sample was isolated using TRIzol reagent (Invitrogen, USA). An RNA Nano 6000 Assay Kit and a Bioanalyzer 2100 system (Agilent Technologies, USA) were employed to determine the integrity of RNA, a Nanodrop instrument (Thermo Fisher Scientific, USA) was used to assess the purity and quantity of RNA, and the RIN ranged from 9.0 to 10.0. Six lncRNA libraries were constructed from SYFs of hens raised under RL (R1, R2, R3) or WL (W1, W2, W3). A total of 3 μg RNA from each sample was used as input material for RNA sample preparation. An Epicentre Ribo-zero rRNA Removal Kit (Epicentre, USA) was used to remove ribosomal RNA, and ethanol precipitation was applied to clean up the rRNA-free samples. An NEBNext Ultra Directional RNA Library Prep Kit for Illumina (NEB, USA) was employed to generate sequencing libraries. Random hexamer primer and M-MuLV Reverse Transcriptase were used to synthesize first-strand cDNA, DNA Polymerase I and RNase H were applied to synthesize second-strand cDNA, and dNTPs with dTTP were replaced by dUTP. The 3′ ends of DNA were adenylated, and NEBNext Adaptor with a hairpin loop structure was ligated to prepare for hybridization. An AMPure XP system (Beckman Coulter, USA) was employed to purify the library fragments. The quality of the library was then measured by an Agilent Bioanalyzer 2100 System. Paired-end reads were sequenced on an Illumina Hiseq 4000 platform (30×) at Shanghai Oebiotech Co., Ltd (Shanghai, China).

### Bioinformatics Analysis

Clean data were obtained by removing adapter sequences, cleaning low-quality tags, and filtering adaptor-ligated contaminants using Trimmomatic v0.38 (Bolger et al., 2014). Reads were then aligned with the chicken genome (http://ftp.ensembl.org/pub/release-76/gtf/gallus_gallus/) using TopHat (Trapnell et al., 2009). Coding-non-coding-index (CNCI ≤ 0), coding potential calculator (CPC ≤ 0), and Pfam (http://pfam.xfam.org) were used to identify the occurrence of any of the known protein family domains documented in the Pfam database (cutoff e-value = e-4), and the polygenetic codon substitution frequency (PhyloCSF score ≤ 20) was applied to distinguish mRNAs from lncRNAs. Potential lncRNA transcripts were compared with the reference annotation file using Cuffcompare to identify novel lncRNAs (Trapnell et al., 2010). Fragments per kilobase of transcript per million mapped reads (FPKM) values were used to estimate the expression levels of the transcripts using the Cuffnorm program (Li et al., 2017). Cuffdiff (v2.1.1) was used to identify differentially expressed lncRNAs and mRNAs (Trapnell et al., 2010), mRNAs, and lncRNAs meeting the criteria of fold change ≥ |2| and q < 0.05.

### Co-expression (trans) and Co-location (cis) Analyses

Pairwise significantly differentially expressed mRNAs and lncRNAs were estimated using the Pearson's correlation coefficient (r). The small number of samples (three each from WL and RL groups) used for the co-expression analysis is a limitation of our study. Those mRNAs with a *p*-value < 0.01 and |r-value| > 0.9 were considered co-expressed genes of their respective lncRNAs. FEELnc (v 0.1.1) (Wucher et al., 2017) was employed to screen the coding genes located within 100 kb upstream and downstream of lncRNAs for potential cis-regulation. RIsearch-2.0 software (Alkan et al., 2017) was used to identify target genes in trans, with parameters set as the base number of direct interactions between lncRNAs and mRNAs ≥10 and free energy ≤-100. Differentially expressed lncRNAs and their corresponding differentially expressed cis- and trans-target genes were used to construct lncRNA-gene interaction networks using Cytoscape v3.2.1 (Smoot et al., 2011).

### Target Gene Prediction

To gain further insight into the functions and classifications of the identified lncRNA targets, we performed Gene Ontology (GO) term and Kyoto Encyclopedia of Genes and Genomes (KEGG) pathway annotation of predicted lncRNA targets using the DAVID gene annotation tool (http://david.abcc.ncifcrf.gov/). We used KOBAS software to test the statistical enrichment of differentially expressed genes and lncRNA target genes in KEGG pathways (Peng et al., 2019).

### Real-Time Quantitative PCR (RT-qPCR) Analysis

Samples were isolated from GCs of SYFs and RT-qPCR was used to validate DE lncRNAs and mRNAs identified by RNA-Seq. RT-qPCR was performed using a LightCycler 480 II Real-time PCR Instrument (Roche, Swiss) with ChamQ SYBR qPCR Master Mix (Vazyme, China). Each 10 μl PCR mixture contained 1 μl of cDNA, 5 μl of 2× ChamQ SYBR qPCR Master Mix, 0.2 μl of forward primer, 0.2 μl of reverse primer, and 3.6 μl of nuclease-free water. Reactions were incubated in a 384-well optical plate (Roche, Switzerland) at 95°C for 30 s, followed by 40 cycles at 95°C for 10 s, and 60°C for 30 s. Each sample was run in triplicate for analysis. At the end of each PCR cycle, melting curve analysis was performed to validate the specific generation of the expected PCR product. Specific primers for mRNAs and lncRNAs are listed in Supplementary Table 1. Using *ACTB* as a reference, relative expression levels of mRNAs and lncRNAs were quantified using the 2^−ΔΔCT^ method (Livak and Schmittgen, 2001).

### Statistical Analysis

Data are expressed as mean ± standard error, and one-way analysis of variance was performed with SPSS 13.0 software (SPSS Inc., Chicago, IL, USA). The statistical significance of differences among the various groups was evaluated by least significant difference *post-hoc* multiple comparisons tests, and *p* < 0.05 was considered statistically significant.

## Results

### Reproductive Performance of Jinghai Yellow Chickens

Egg production by hens aged 300 days in the RL and WL groups is shown in Table 1. Egg production in the RL group was significantly greater than that in the control group (*p* = 0.023), but there was not statistically significant difference in egg weight between groups (*p* = 0.667). Although the body weight of hens was slightly higher in the RL group (*p* = 0.925), the difference was not statistically significant. These results suggest that RL enhanced the reproductive performance of hens.

**Table 1:** Reproductive performance of hens at age of 300 days under monochromatic lights.

**Treatment group**	**Egg production**	**Egg weight**	**Body weight**
WL	87.44 ± 0.93^a^	50.49 ± 0.41	2150.4 ± 21.44
RL	90.61 ± 1.01^b^	50.72 ± 0.36	2102.3 ± 4.39

a, b *Mean ± standard error. Different superscripts indicate significantly different values (p < 0.05)*.

### Identification of lncRNAs and mRNAs in Hen Ovaries

**Figure 1:**
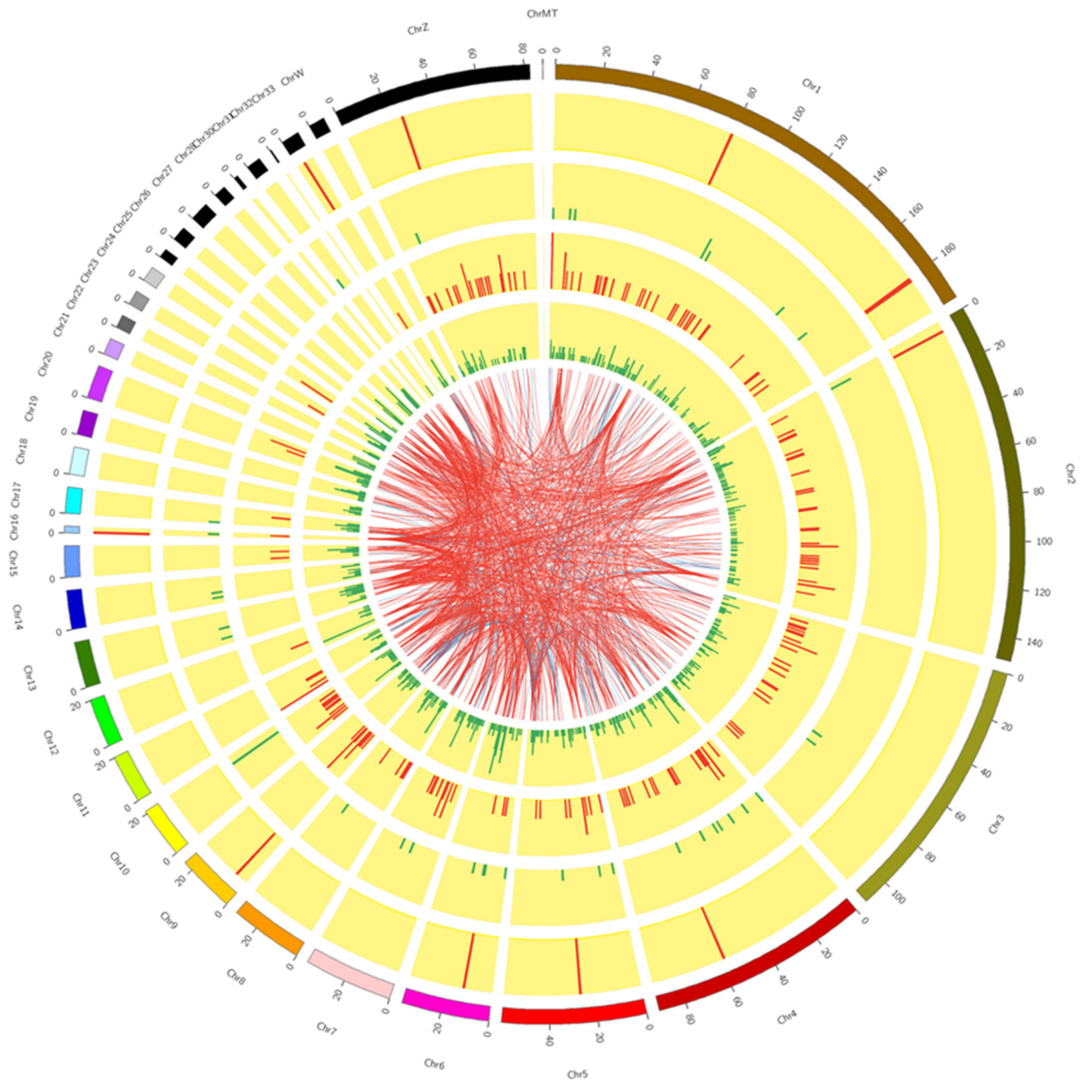
Circos plot overview of lncRNA sequencing data. The inner plots show chromosomes 1 to 32 shared between GCs from SYFs in the RL and WL groups.

Six cDNA libraries were built from the RL (*n* = 3) and WL (*n* = 3) groups to identify lncRNAs and mRNAs expressed in GCs of SYFs. We obtained 97.97–99.10 million raw reads after filtering out contaminated reads, low-quality reads, and those with unknown bases accounting for >5% of reads, resulting in 90.45–95.06 million clean reads (Supplementary Table 2). Next, 87.66–91.81% of clean reads from each library were mapped to the chicken reference genome. The average GC content was 47.81%, and Circos analysis showed that lncRNAs in GCs were distributed on almost all chromosomes, with the fewest on chromosome 32 and the most on chromosome 1 (Figure 1).

**Figure 2:**
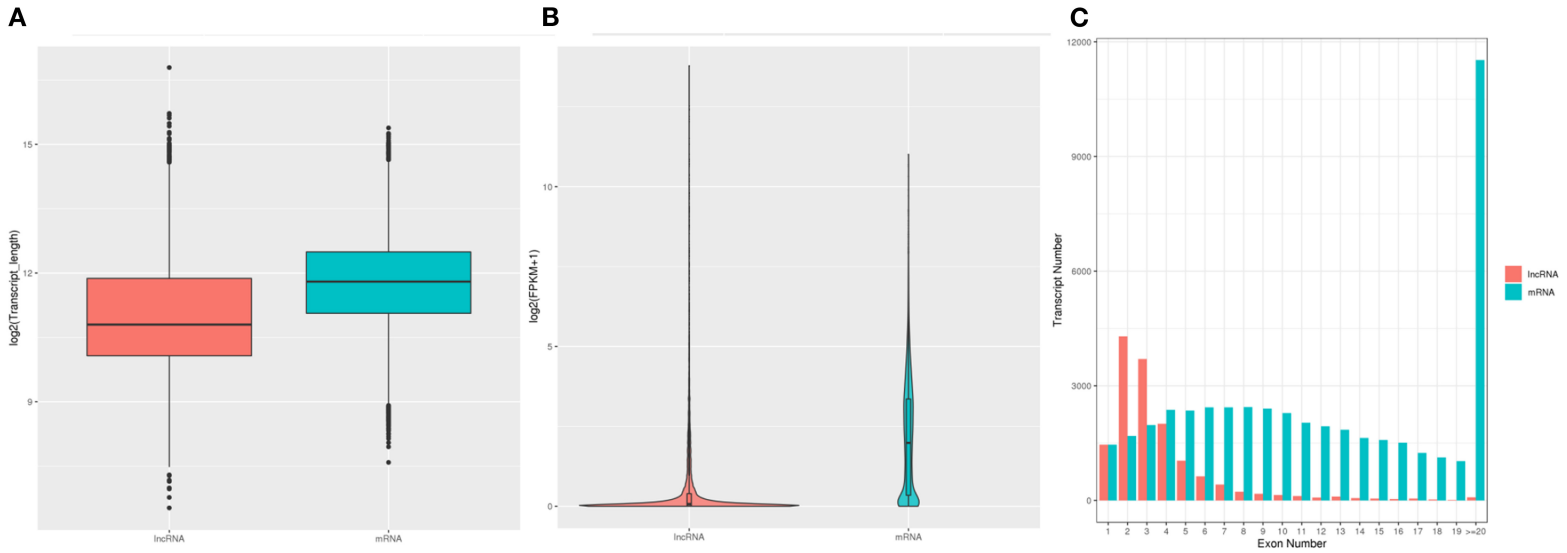
Distribution of transcript length, number of exons, and expression levels of lncRNAs and mRNAs in GCs of SYFs from hens in RL and WL groups. **(A)** Distribution of the transcript lengths of identified lncRNAs and mRNAs. **(B)** Expression distribution of the identified lncRNAs and mRNA transcripts. **(C)** Distribution of the number of exons of lncRNA and mRNA transcripts.

A stringent filtering pipeline was applied to discard transcripts lacking all lncRNA characteristics, transcripts <200 bp in length, and those with only two exons and three reads of coverage. The lncRNA genes had an average length of 1,408 bp and 2.5 exons. A total of 12,466 lncRNAs were included in the assembled transcripts, comprising 10,969 and 1,497 known and unknown lncRNAs (Supplementary Table 3). The majority of lncRNAs were from the genic intronic region (Supplementary Table 3). Expression levels, transcript lengths, and the number of exons between lncRNAs and mRNAs generated from six individual chicken samples are shown in Figure 2. The length of mRNA transcripts was greater than the length of lncRNAs, and most mRNAs included more than 20 exons, compared with only two or three exons in most lncRNAs. Furthermore, the average expression level measured for lncRNAs was significantly lower than that of mRNAs.

### Analysis of Differentially Expressed lncRNAs

**Figure 3:**
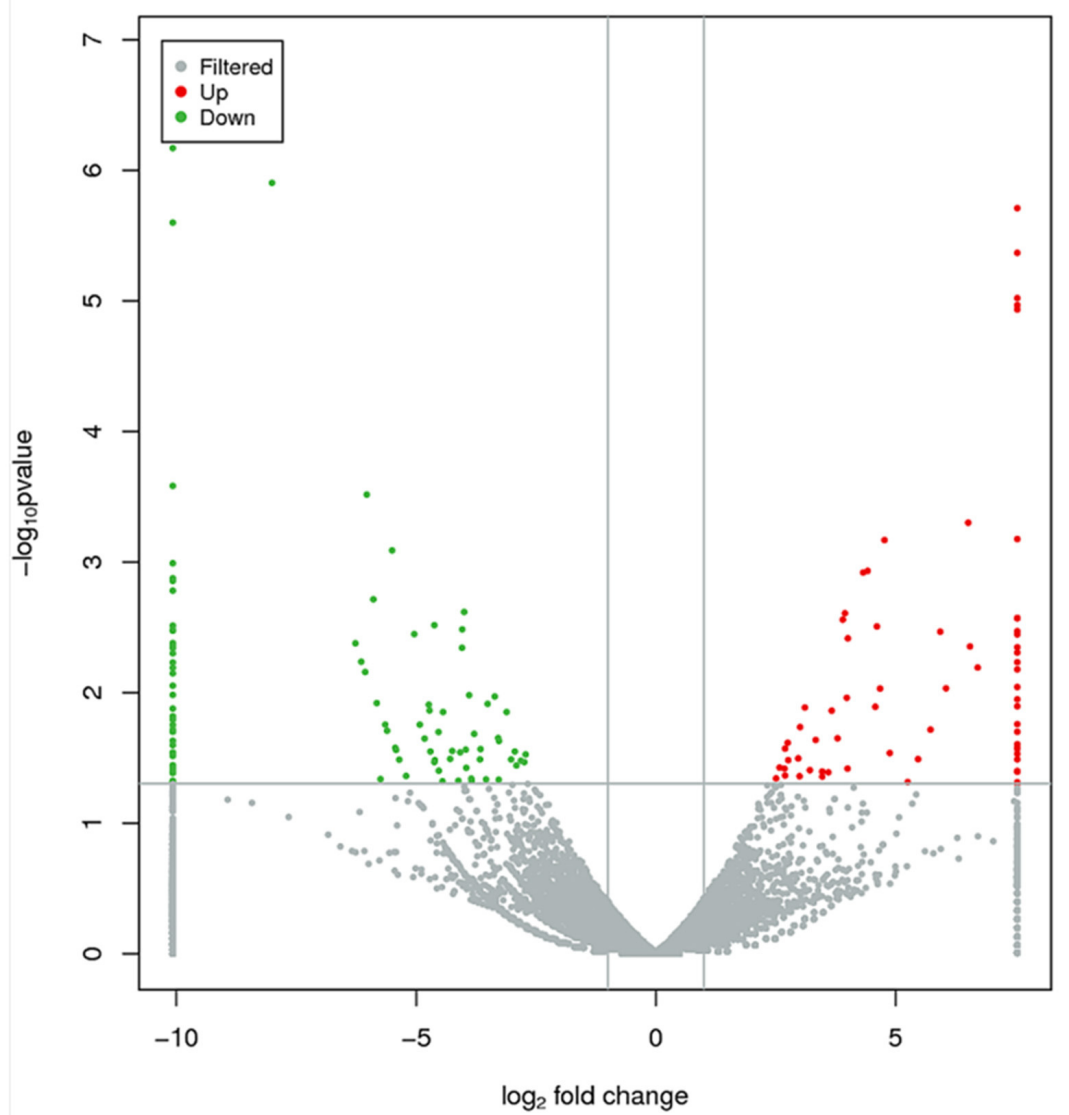
Differentially expressed lncRNAs between RL and WL groups. Red and green dots represent up- and downregulated lncRNAs, respectively. Gray dots represent lncRNAs without significant differential expression.

**Figure 4:**
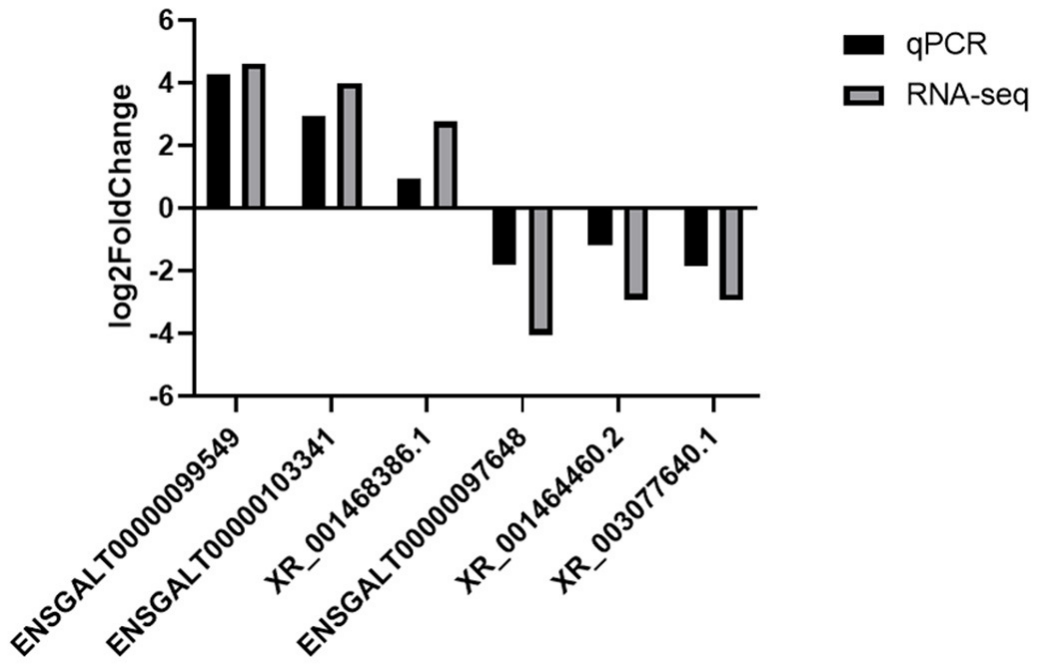
Validation of differentially expressed lncRNAs by RT-qPCR.

**Figure 5:**
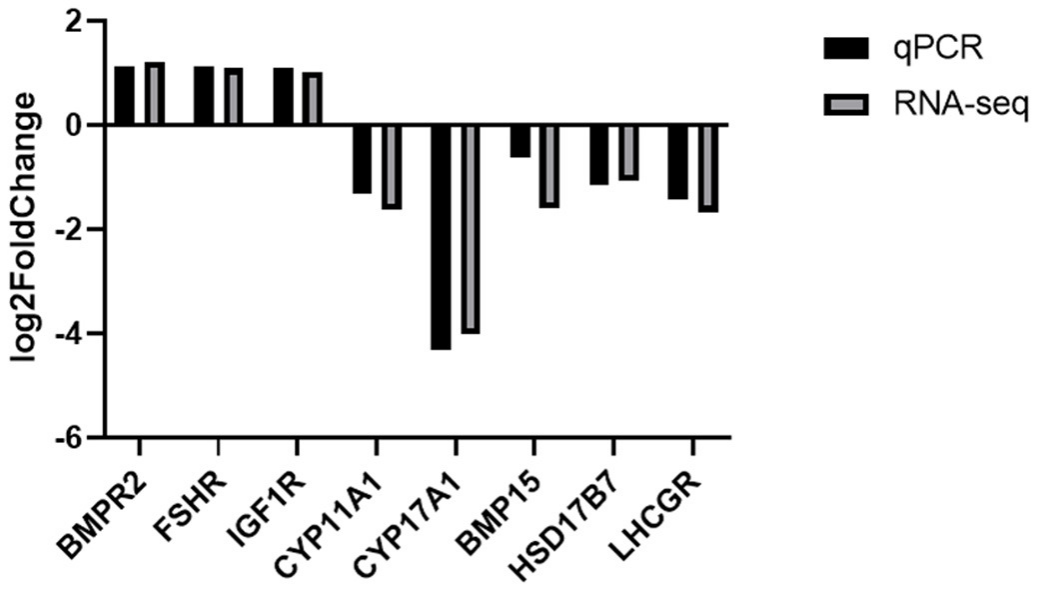
Validation of the expression levels of lncRNA target mRNAs between RL and WL groups.

Based on the cutoff criteria for distinguishing differentially expressed lncRNAs, 168 lncRNAs were significantly different between RL and WL groups (*p* < 0.05), of which 101 were downregulated and 67 were upregulated in the RL group (Figure 3, Supplementary Table 4). Furthermore, 1,182 differentially expressed mRNAs were identified (958 downregulated and 224 upregulated). RT-qPCR was applied to validate the RNA-Seq results using six candidate lncRNAs and eight mRNAs; cytochrome P450 family 11 subfamily A member 1 (*CYP11A1*), cytochrome P450 17A1 (*CYP17A1*), 7β-hydroxysteroid dehydrogenase type 7 (*HSD17B7*), bone morphogenetic protein 15 (*BMP15*), bone morphogenetic protein receptor 2 (*BMPR2*), luteinizing hormone/choriogonadotropin receptor (*LHCGR*), follicle stimulating hormone receptor (*FSHR*), and insulin-like growth factor 1 receptor (*IGF1R*). The results showed that the expression patterns of these mRNAs and lncRNAs were similar to the results of high-throughput sequencing (Figures 4, 5), indicating that the RNA-Seq data were reliable.

### Interactions Between lncRNAs and mRNAs Involved in Reproduction

**Figure 6:**
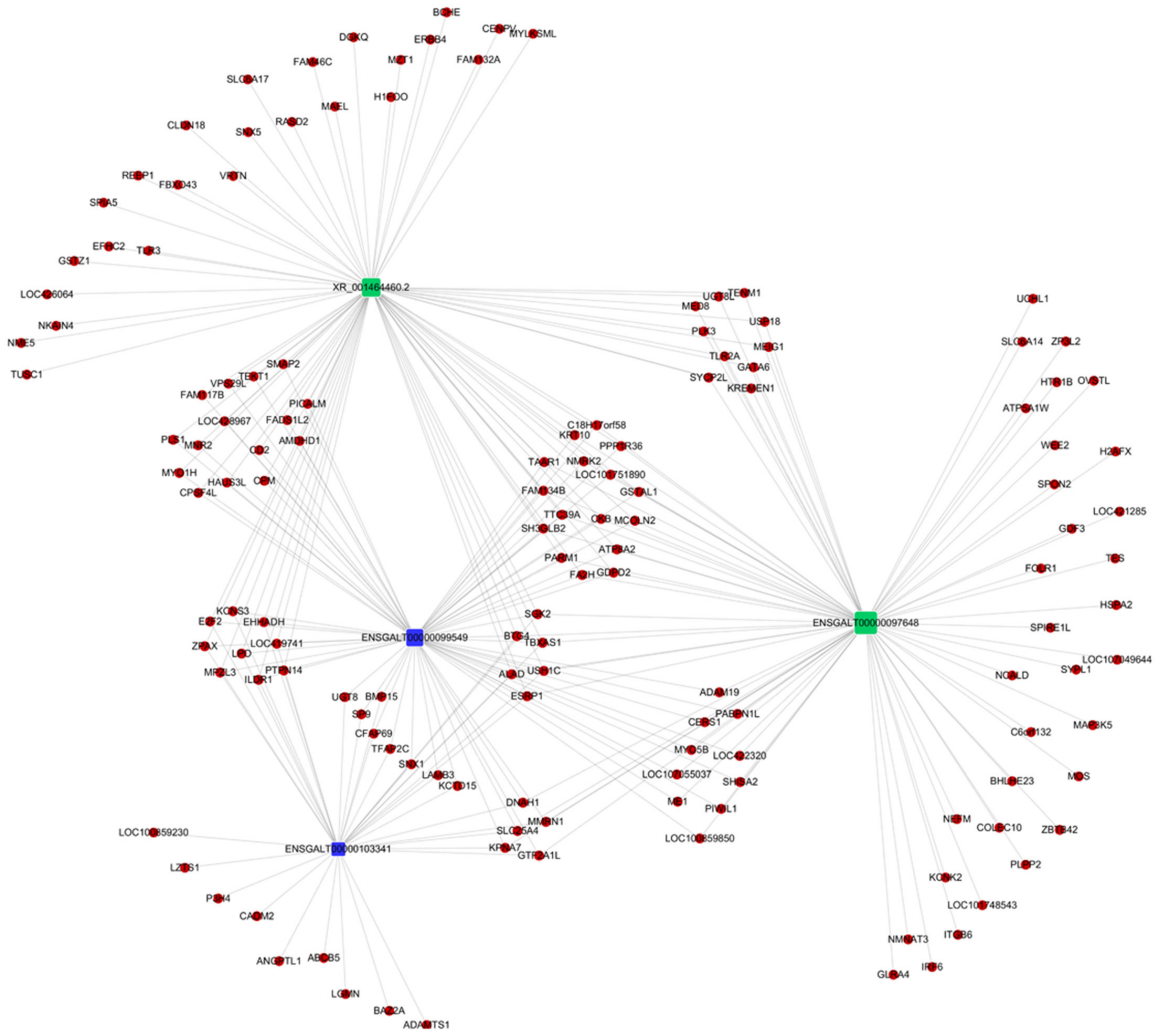
Regulatory network of four differentially expressed lncRNAs involved in regulating follicular development in Jinghai Yellow chickens under monochromatic light.

**Figure 7:**
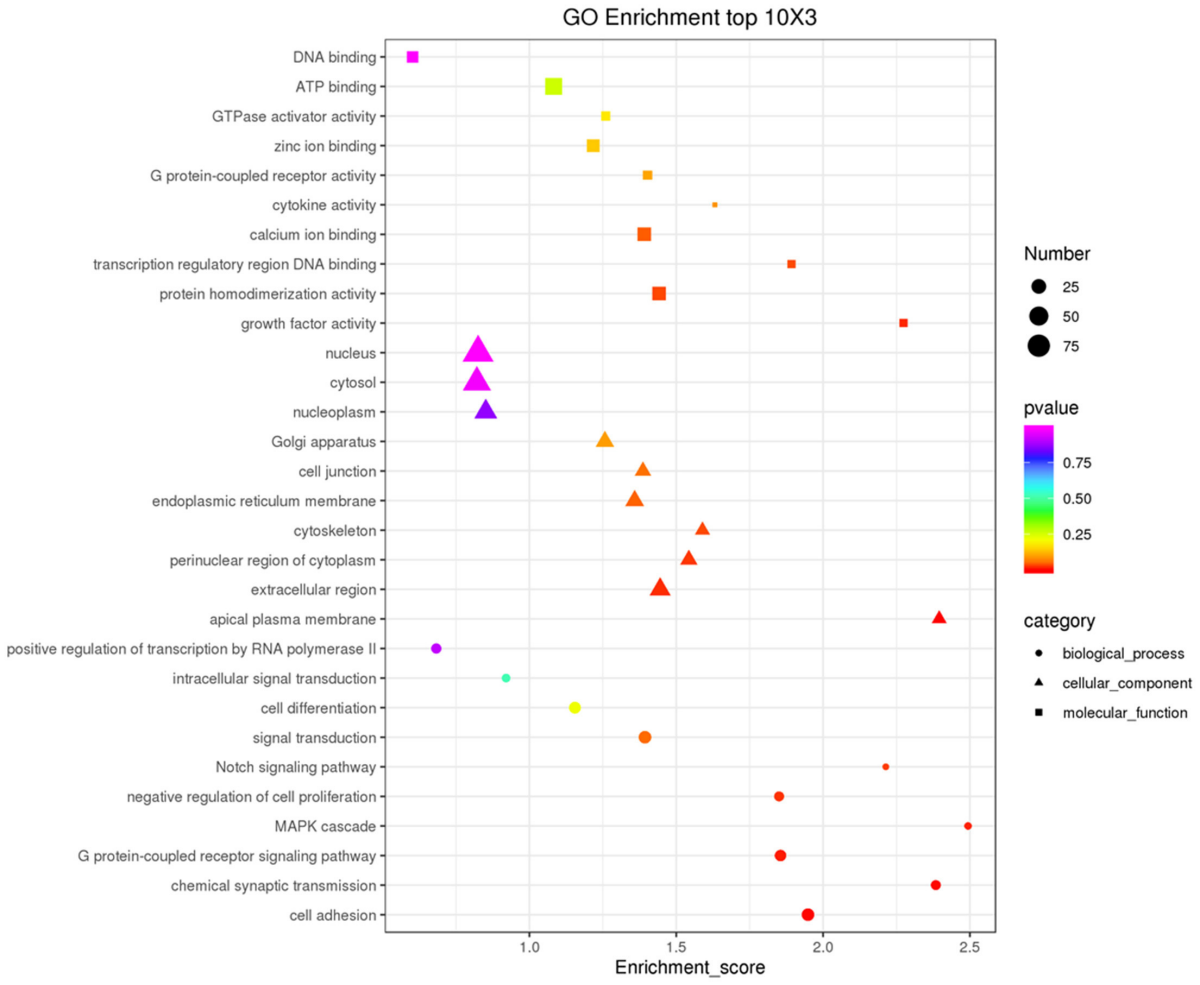
GO analysis of host genes of differentially expressed lncRNAs. The top 10 GO enrichment terms in Biological Process (BP), Cellular Component (CC), and Molecular Function (MF) categories are included for target mRNAs of all differentially expressed lncRNAs.

**Figure 8:**
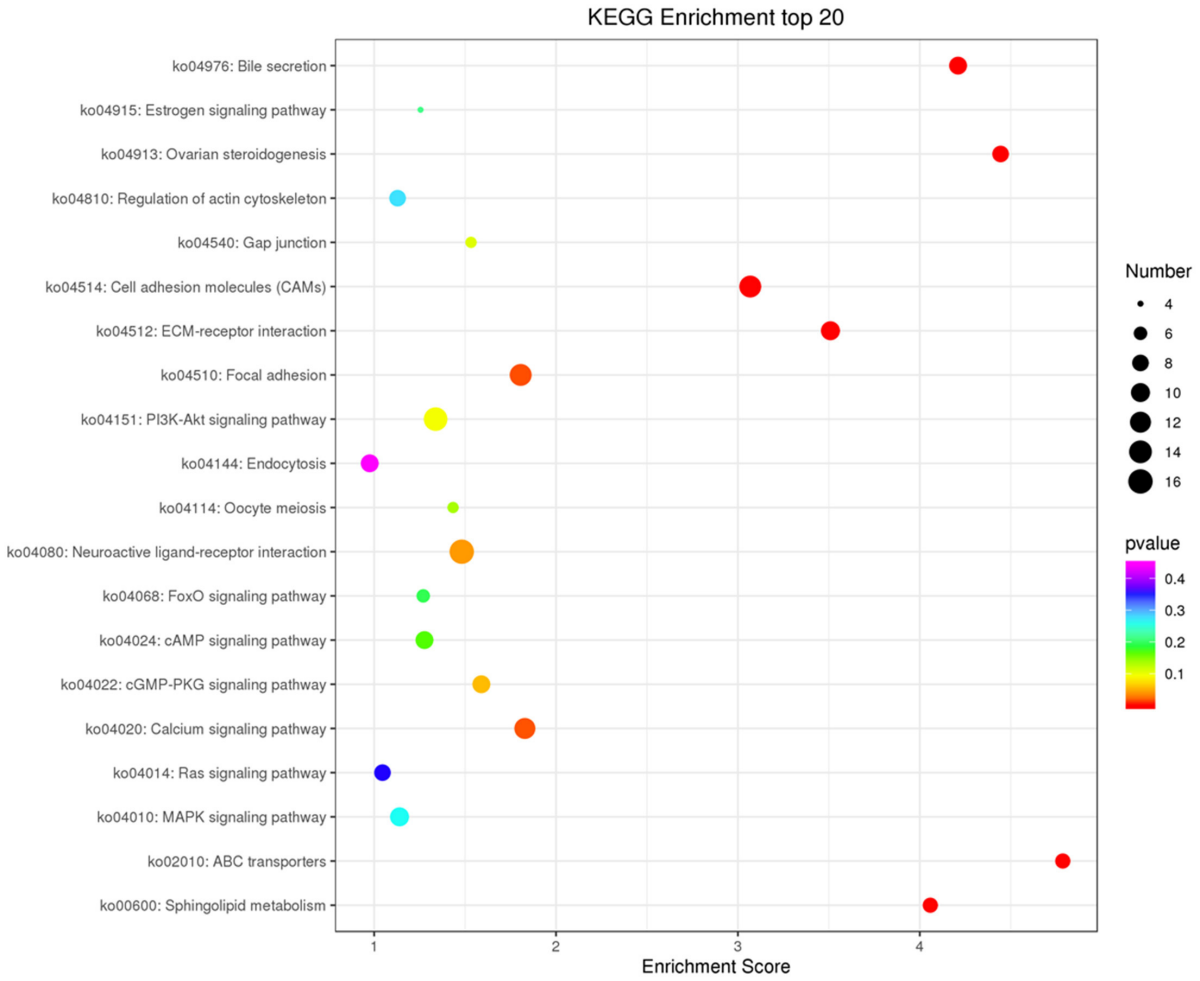
KEGG analysis of target mRNAs of differentially expressed lncRNAs. The top 20 KEGG enrichment terms in Biological Process (BP), Cellular Component (CC), and Molecular Function (MF) categories are included for target mRNAs of all differentially expressed lncRNAs.

Co-expression of different lncRNAs and mRNAs was analyzed using Pearson correlation tests to calculate expression correlations between differentially expressed lncRNAs (length <6000 bp) and differentially expressed mRNAs. Correlation coefficient ≥ 0.9 and *p*-value ≤ 0.01 were selected as the cutoff criteria, and 120 lncRNAs targeting 1,175 mRNAs were identified (Supplementary Table 5). Potential cis and trans targets of lncRNAs were predicted to explore the functions of lncRNAs. Nine lncRNAs were identified with cis functions and 39 lncRNAs were predicted with trans functions. Four lncRNAs (ENSGALT00000099549, ENSGALT00000103341, ENSGALT00000097648, and XR_001464460.2) were predicted to target 344 mRNAs (Figure 6, Supplementary Table 6). ENSGALT00000099549, ENSGALT00000097648, and XR_001464460.2 target a set of mRNAs related to ovarian steroidogenesis, including *CYP11A1, BMP15, HSD17B7*, and *LHCGR*. ENSGALT00000103341 targets a set of mRNAs related to sphingolipid metabolism, including ABC transporters such as *UGT8, UGT8L, ABCB5, ABCA4*, and *ABCG2*. XR_003077640.1 regulates genes such as *CYP17A1, IGF1R*, and *BMPR2*, while XR_001468386.1 regulates genes including *PIK3R1, PAK5, SEMA3C*, and *BMPR2*. Furthermore, the target mRNAs of these lncRNAs were enriched in various GO functions including negative regulation of cell proliferation, cell differentiation, and the Notch signaling pathway (Figure 7). Meanwhile, pathway enrichment analyses identified ovarian steroidogenesis, cell adhesion molecules, and ABC transporters (Figure 8).

## Discussion

Our results demonstrate that monochromatic light affects the egg laying performance of Jinghai Yellow chickens. Compared with WL, RL improved egg production, in agreement with a previous study on hens of Pyrzak et al. (1986). Foster and Follett (1985) reported that RL elicits a greater photosexual response than the same photon flux of other wavelengths, because longer wavelengths penetrate more easily to reach the hypothalamus. Pyrzak and Siopes (1986) suggested that RL increases egg weight, but our results showed that RL had only a minor effect on the egg weight of chickens. Specifically, the body weight of chickens at 300 days of age under RL was similar to that under WL, consistent with Li et al. (2014). RL can clearly improve the egg laying performance of chickens, but the molecular mechanisms associated with the effects of monochromatic light remain obscure.

We compared the lncRNA expression profiles of GCs from SYFs of Jinghai Yellow hens under RL and WL, and identified a number of lncRNA target mRNAs related to egg production. Compared with the WL group, 168 lncRNAs were differentially expressed in the RL group (101 downregulated and 67 upregulated). Target prediction and functional analysis of these genes, including ENSGALT00000099549, ENSGALT00000103341, ENSGALT00000097648, XR_001468386.1, XR_003077640.1, and XR_001464460.2, showed that many lncRNA target mRNAs were associated with steroid hormone biosynthesis, ovarian steroidogenesis, TGF-β signaling pathway, prolactin signaling pathway, MAPK signaling pathway, estrogen signaling pathway, PI3K-Akt signaling pathway, Hippo signaling pathway, MAPK signaling pathway, and cell adhesion molecules. In our previous studies, steroid hormone biosynthesis and the PI3K-Akt signaling pathway were also altered in pigeons under monochromatic light (Wang et al., 2015, 2019). Huang et al. (1979) suggested that progesterone is synthesized by GCs, and Hu et al. (2017) concluded that PI3K and AMPK signaling pathways converge to modulate ERK activity, and thereby regulate GC differentiation. Meanwhile, Kim et al. (2013) showed that the TGF-β signaling pathway is involved in initiating follicle selection, in accordance with our current results, suggesting that these pathways play a key role in follicular development under monochromatic light.

Eight overlapping targets identified by RNA-Seq were selected for verification by RT-qPCR, including *CYP11A1, CYP17A1*, and *17*β*-HSD*, which are important for steroidogenesis and the synthesis of hormones such as testosterone, progesterone, and estrogen (Zhou et al., 2020). P450scc, the protein product of the *CYP11A1* gene, plays a major role in the control of steroidogenesis (Simpson, 1979; Hara et al., 2014). In chicken follicular GCs, expression of *CYP11A1* is a prerequisite for progesterone synthesis, and is related to follicle selection. Nebert et al. (2013) reported that *CYP11A1* knockout mice display a variety of aberrant phenotypes associated with various steroid hormone deficiency syndromes. *CYP11A1* and *CYP17A1* have the capacity to synthesize androgenic substrates that diffuse into adjacent pre-granulosa cells (Lagaly et al., 2008). *HSD17B7* converts estrone to estradiol, *HSD17B7* is involved in cholesterol biosynthesis and has 3-ketosteroid reductase activity (Nokelainen et al., 1998; Shehu et al., 2008), and mouse *HSD17B7*^−/−^ fetuses lack proteins involved in cholesterol synthesis (Saher et al., 2005). Compared with large follicles, GCs of small follicles have lower *CYP11A1* levels. Hatzirodos et al. (2014) also pointed out that early in follicle development, the hormone inhibin is secreted, and oestradiol is later secreted at the preovulatory stage, which may explain why these genes were down-regulated in the RL group in the present work.

Follicular development and differentiation of GCs are dependent on regulation of LH and FSH, and their specific receptors (Richards et al., 1976). LH induces progesterone secretion through *LHCGR*, and an increase in granulosa *LHCGR* causes a progressive increase in the responsiveness of GCs to LH in maturing follicles (Bahr and Johnson, 1984). *LHCGR* is differentially regulated between small and large follicles (Peng et al., 1991), and is downregulated in small follicles (Hatzirodos et al., 2014), in accordance with our current results. Recent studies have shown that all SYFs isolated from the same ovary in a laying hen can express *FSHR*, and respond to stimulation by FSH (Webb et al., 2003; Johnson et al., 2015). One of the earliest markers for differentiating GCs is elevated expression of *FSHR* mRNA, specifically within the granulosa layer (Johnson and Woods, 2009). In hens, SYFs expressing the highest levels of *FSHR* are recruited into the preovulatory hierarchy during ovarian follicle development (Woods and Johnson, 2005; Wang et al., 2017). Herein, GCs of SYFs in the RL group had higher *FSHR* levels, indicating more preovulatory hierarchy follicles.

As members of the TGF-β superfamily, *BMPs* and their receptors have been shown to play important roles during folliculogenesis (Juengel and McNatty, 2005). *BMP15* promotes follicle selection in hens (Stephens and Johnson, 2016). Moore et al. (2003) demonstrated that *BMP15* promotes mitosis of GCs and suppresses *FSHR* expression, leading to the suppression of FSH-induced progesterone synthesis, and stimulation of kit ligand expression, in accordance with the results of the present study. *FSHR* was upregulated and *BMP15* was downregulated in the RL group. *BMPR2* binds *GDF-9* and *BMP-15* (Hatzirodos et al., 2014), and dysregulation of *BMPR2* gene expression has been associated with abnormalities in follicular development (Andreas et al., 2016). *In vivo*, GCs require *IGF1R* to undergo differentiation upon FSH stimulation (Zhou et al., 2013), and upregulation of *IGF1R* in the RL group is in accordance with FSH. Baumgarten et al. (2017) concluded that *IGFR* is necessary for the formation of preovulatory follicles and the differentiation of GCs to the preovulatory stage.

In conclusion, we found that RL enhanced the egg production of hens. High-throughput sequencing identified a set of differentially expressed lncRNAs and mRNAs in GCs of SYFs between RL and WL groups. The results provide a starting point for studies aimed at understanding the molecular mechanisms by which red light increases the egg production of Jinghai Yellow chickens. Furthermore, the findings will facilitate research on the effects of red light on follicular development in Jinghai Yellow chickens and other poultry.

## Data Availability Statement

The datasets presented in this study can be found in online repositories. The names of the repository/repositories and accession number(s) can be found below: NCBI Sequence Read Archive (SRP278036), BioProject (PRJNA657681).

## Ethics Statement

The animal study was reviewed and approved by Institutional Animal Care and Use Committee of the Department of Animal Science and Technology, Yangzhou University, China.

## Author Contributions

YW, JW, and GZ contributed to the overall design of the study. YW, HS, and YG collected data. YW, HS, MS, TL, XL, and LC contributed to sample collection. YW participated in manuscript writing and revision. All authors approved the final version of the manuscript.

## Conflict of Interest

HS and YG were employed by the company Jiangsu Jinghai Poultry Industry Group Co. Ltd. The remaining authors declare that the research was conducted in the absence of any commercial or financial relationships that could be construed as a potential conflict of interest.

## References

[B1] Alkan F. Wenzel A. Palasca O. Kerpedjiev P. Rudebeck A. F. Stadler P. F.. (2017). RIsearch2: suffix array-based large-scale prediction of RNA–RNA interactions and siRNA off-targets. Nucleic Acids Res. 45:60. 10.1093/nar/gkw1325 28108657 PMC5416843

[B2] Andreas E. Hoelker M. Neuhoff C. Tholen E. Schellander K. Tesfaye D.. (2016). MicroRNA 17-92 cluster regulates proliferation and differentiation of bovine granulosa cells by targeting PTEN and BMPR2 genes. Cell Tissue Res. 366, 219–230. 10.1007/s00441-016-2425-7 27221279

[B3] Bahr J. M. Johnson A. L. (1984). Regulation of the follicular hierarchy and ovulation. J. Exp. Zool. 232, 495–500. 10.1002/jez.1402320316 6097631

[B4] Baumgarten S. C. Armouti M. Ko C. Stocco C. (2017). IGF1R expression in ovarian granulosa cells is essential for steroidogenesis, follicle survival, and fertility in female mice. Endocrinology. 158, 2309–2318. 10.1210/en.2017-00146 28407051 PMC5505221

[B5] Bolger A. M. Marc L. Bjoern U. (2014). Trimmomatic: A flexible trimmer for Illumina sequence data. Bioinformatics. 30, 2114–2120. 10.1093/bioinformatics/btu170 24695404 PMC4103590

[B6] Casey J. M. Harrison P. C. Latshaw J. D. McGinnis J. (1969). Effects of photoperiod and colored lights on the sexual maturity of domestic fowl. Poult. Sci. 48:1794.

[B7] Cheetham S. W. Gruhl F. Mattick J. S. Dinger M. E. (2013). Long noncoding RNAs and the genetics of cancer. Br. J. Cancer. 108, 2419–2425. 10.1038/bjc.2013.233 23660942 PMC3694235

[B8] Foss D. C. White J. L. (1983). Early sexual maturity of brown-egg pullets cage-grown in narrowband light with high nutrient density diets. Poult. Sci. 62:1424.

[B9] Foster R. G. Follett B. K. (1985). The involvement of a rhodopsin-like photopigment in the photoperiodic response of the Japanese quail. J. Comp. Physiol. A. 157, 519–528. 10.1007/BF00615153

[B10] Gilbert A. B. Evans A. J. Perry M. M. Davidson M. H. (1977). A method for separating the granulosa cells, the basal lamina and the theca of the preovulatory ovarian follicle of the domestic fowl (Gallus domesticus). J. Reprod. Fertil. 50, 179–181. 10.1530/jrf.0.0500179 864645

[B11] Gongruttananun N. (2011). Influence of red light on reproductive performance, eggshell ultrastructure, and eye morphology in Thai-native hens. Poult. Sci. 90, 2855–2863. 10.3382/ps.2011-01652 22080025

[B12] Hara L. York J. P. Zhang P. Smith L. B. (2014). Targeting of GFP-Cre to the mouse Cyp11a1 locus both drives cre recombinase expression in steroidogenic cells and permits generation of Cyp11a1 knock out mice. PLoS ONE 9:e84541. 10.1371/journal.pone.0084541 24404170 PMC3880310

[B13] Hatzirodos N. Irving-Rodgers H. F. Hummitzsch K. Harland M. L. Morris S. E. Rodgers R. J. (2014). Transcriptome profiling of granulosa cells of bovine ovarian follicles during growth from small to large antral sizes. BMC Gen. 15:24. 10.1186/1471-2164-15-24 24422759 PMC3898003

[B14] Hernandez A. G. Bahr J. M. (2003). Role of FSH and epidermal growth factor (EGF) in the initiation of steroidogenesis in granulosa cells associated with follicular selection in chicken ovaries. Reproduction. 125, 683–691. 10.1530/rep.0.1250683 12713431

[B15] Hsueh A. J. Adashi E. Y. Jones P. B. Welsh Jr T. H. (1984). Hormonal regulation of the differentiation of cultured ovarian granulosa cells. Endocr.Rev. 5, 76–127. 10.1210/edrv-5-1-76 6142819

[B16] Hu S. Duggavathi R. Zadworny D. (2017). Regulatory mechanisms underlying the expression of prolactin receptor in chicken granulosa cells. PLoS ONE 12:e0170409. 10.1371/journal.pone.0170409 28107515 PMC5249103

[B17] Huang E. S. R. Kao K. J. Nalbandov A. V. (1979). Synthesis of sex steroids by cellular components of chicken follicles. Biol. Reprod. 20, 454–461. 10.1095/biolreprod20.3.454 454748

[B18] Johnson A. L. Woods D. C. (2009). Dynamics of avian ovarian follicle development: cellular mechanisms of granulosa cell differentiation. Gen. Comp. Endocrinol. 163, 12–17. 10.1016/j.ygcen.2008.11.012 19059411

[B19] Johnson P. A. Stephens C. S. Giles J. R. (2015). The domestic chicken: causes and consequences of an egg a day. Poult. Sci. 94, 816–820. 10.3382/ps/peu083 25667424

[B20] Jones J. E. Hughes B. L. Thurston R. J. Hess R. A. Froman D. P. (1982). The effects of red and white light during the prebreeder and breeder periods on egg production and feed consumption in large white turkeys. Poult. Sci. 61, 1930–1932. 10.3382/ps.0611930 7134148

[B21] Juengel J. L. McNatty K. P. (2005). The role of proteins of the transforming growth factor-beta superfamily in the intraovarian regulation of follicular development. Hum. Reprod. Update. 11, 143–160. 10.1093/humupd/dmh061 15705960

[B22] Kim D. Ocón-Grove O. Johnson A. L. (2013). Bone morphogenetic protein 4 supports the initial differentiation of hen (*Gallus gallus*) granulosa cells. Biol. Reprod. 88, 1–7. 10.1095/biolreprod.113.109694 23658430

[B23] La Y. He X. Zhang L. Di R. Wang X. Gan S.. (2020). Comprehensive analysis of differentially expressed profiles of mRNA, lncRNA, and circRNA in the uterus of seasonal reproduction sheep. Genes. 11:301. 10.3390/genes11030301 32178360 PMC7140836

[B24] Lagaly D. V. Aad P. Y. Grado-Ahuir J. A. Hulsey L. B. Spicer L. J. (2008). Role of adiponectin in regulating ovarian theca and granulosa cell function. Mol. Cell Endocrinol. 284, 38–45. 10.1016/j.mce.2008.01.007 18289773

[B25] Li D. Zhang L. Yang M. Yin H. Xu H. Trask J. S. (2014). The effect of monochromatic light-emitting diode light on reproductive traits of laying hens. J. Appl. Poult. Res. 23, 367–375. 10.3382/japr.2013-00746

[B26] Li Z. Ouyang H. Zheng M. Cai B. Han P. Abdalla B. A.. (2017). Integrated analysis of long non-coding RNAs (LncRNAs) and mRNA expression profiles reveals the potential role of LncRNAs in skeletal muscle development of the chicken. Front Physiol. 7:687. 10.3389/fphys.2016.00687 28119630 PMC5220077

[B27] Liu Y. Qi B. Xie J. Wu X. Ling Y. Cao X.. (2018). Filtered reproductive long non-coding RNAs by genome-wide analyses of goat ovary at different estrus periods. BMC Gen. 19, 1–13. 10.1186/s12864-018-5268-7 30509164 PMC6278114

[B28] Livak K. J. Schmittgen T. D. (2001). Analysis of relative gene expression data using real-time quantitative PCR and the 2^−ΔΔCT^ method. Methods. 25, 402–408. 10.1006/meth.2001.1262 11846609

[B29] Moore R. K. Otsuka F. Shimasaki S. (2003). Molecular basis of bone morphogenetic protein-15 signaling in granulosa cells. J. Biol. Chem. 278, 304–310. 10.1074/jbc.M207362200 12419820

[B30] Nebert D. W. Wikvall K. Miller W. L. (2013). Human cytochromes P450 in health and disease. Philos. Trans. R. Soc. Lond B. Biol. Sci. 368:20120431. 10.1098/rstb.2012.0431 23297354 PMC3538421

[B31] Nokelainen P. Peltoketo H. Vihko R. Vihko P. (1998). Expression cloning of a novel estrogenic mouse 17β-hydroxysteroid dehydrogenase/17-ketosteroid reductase (m17HSD7), previously described as a prolactin receptor-associated protein (PRAP) in rat. Mol. Endocrinol. 12, 1048–1059. 10.1210/mend.12.7.0134 9658408

[B32] Onagbesan O. Bruggeman V. Decuypere E. (2009). Intra-ovarian growth factors regulating ovarian function in avian species: a review. Anim. Reprod. Sci. 111, 121–140. 10.1016/j.anireprosci.2008.09.017 19028031

[B33] Peng X. R. Hsueh A. J. LaPolt P. S. Bjersing L. Ny T. (1991). Localization of luteinizing hormone receptor messenger ribonucleic acid expression in ovarian cell types during follicle development and ovulation. Endocrinology. 129, 3200–3207. 10.1210/endo-129-6-3200 1954899

[B34] Peng Y. Chang L. Wang Y. Wang R. Hu L. Zhao Z.. (2019). Genome-wide differential expression of long noncoding RNAs and mRNAs in ovarian follicles of two different chicken breeds. Genomics. 111, 1395–1403. 10.1016/j.ygeno.2018.09.012 30268779

[B35] Pyrzak R. Siopes T. D. (1986). Effect of light quality on egg production of caged turkey hens. Poult. Sci. 65, 199–200. 10.3382/ps.0651262

[B36] Pyrzak R. Snapir N. Goodman G. Arnon E. Perek M. (1986). The influence of light quality on initiation of egg laying by hens. Poult. Sci. 65, 90–193. 10.3382/ps.0650190

[B37] Pyrzak R. Snapir N. Goodman G. Perek M. (1984). The influence of light quality on egg production and egg quality of the domestic hen. Poult. Sci. 63:30.

[B38] Ren J. Du X. Zeng T. Chen L. Shen J. Lu L.. (2017). Divergently expressed gene identification and interaction prediction of long noncoding RNA and mRNA involved in duck reproduction. Anim. Reprod. Sci. 185, 8–17. 10.1016/j.anireprosci.2017.07.012 28886878

[B39] Richards J. S. Ireland J. J. Rao M. C. Bernath G. A. Midgley J. R. A. R.. (1976). Ovarian follicular development in the rat: hormone receptor regulation by estradiol, follicle stimulating hormone and luteinizing hormone. Endocrinology. 99, 1562–1570. 10.1210/endo-99-6-1562 187412

[B40] Robinson F. E. Etches R. J. (1986). Ovarian steroidogenesis during foillicular maturation in the domestic fowl (*Gallus domesticus*). Biol. Reprod. 35, 1096–1105. 10.1095/biolreprod35.5.1096 2950935

[B41] Saher G. Brügger B. Lappe-Siefke C. Möbius W. Tozawa R. I. Wehr M. C.. (2005). High cholesterol level is essential for myelin membrane growth. Nat. Neurosci. 8, 468–475. 10.1038/nn1426 15793579

[B42] Schumaier G. Harrison P. C. McGinnis J. (1968). Effect of colored fluorescent light on growth, cannibalism, and subsequent egg production of single comb white leghorn pullets. Poult. Sci. 47, 1599–1602. 10.3382/ps.0471599 5728232

[B43] Shehu A. Mao J. Gibori G. B. Halperin J. Le J. Sangeeta Devi Y.. (2008). Prolactin receptor-associated protein/17β-hydroxysteroid dehydrogenase type 7 gene (Hsd17b7) plays a crucial role in embryonic development and fetal survival. Mol. Endocrinol. 22, 2268–2277. 10.1210/me.2008-0165 18669642 PMC2582539

[B44] Simpson E. R. (1979). Cholesterol side-chain cleavage, cytochrome P450, and the control of steroidogenesis. Mol. Cell. Endocrinol. 13, 213–227. 10.1016/0303-7207(79)90082-0 221289

[B45] Smoot M. E. Ono K. Ruscheinski J. Wang P. L. Ideker T. (2011). Cytoscape 2.8: new features for data integration and network visualization. Bioinformatics. 27, 431–432. 10.1093/bioinformatics/btq675 21149340 PMC3031041

[B46] Stephens C. S. Johnson P. A. (2016). Bone morphogenetic protein 15 may promote follicle selection in the hen. Gen. Comp. Endocrinol. 235, 170–176. 10.1016/j.ygcen.2016.06.027 27340039

[B47] Trapnell C. Pachter L. Salzberg S. L. (2009). TopHat: discovering splice junctions with RNA-Seq. Bioinformatics. 25, 1105–1111. 10.1093/bioinformatics/btp120 19289445 PMC2672628

[B48] Trapnell C. Williams B. A. Pertea G. Mortazavi A. Kwan G. Van Baren M. J.. (2010). Transcript assembly and quantification by RNA-Seq reveals unannotated transcripts and isoform switching during cell differentiation. Nat. Biotechnol. 28, 511–515. 10.1038/nbt.1621 20436464 PMC3146043

[B49] Wang Y. Chen Q. Liu Z. Guo X. Du Y. Yuan Z.. (2017). Transcriptome analysis on single small yellow follicles reveals that Wnt4 is involved in chicken follicle selection. Front. Endocrinol. 8:317. 10.3389/fendo.2017.00317 29187833 PMC5694752

[B50] Wang Y. Ding J. T. Yang H. M. Yan Z. J. Cao W. Li Y. B. (2015). Analysis of pigeon (Columba) ovary transcriptomes to identify genes involved in blue light regulation. PLoS ONE 10:e0143568. 10.1371/journal.pone.0143568 26599806 PMC4657987

[B51] Wang Y. Li Y. B. Yang H. M. Wang Z. Y. (2018). Effect of monochromatic lights on egg production, sex hormone levels, and expression of their receptors in pigeons. Livest. Sci. 216, 233–236. 10.1016/j.livsci.2018.09.005

[B52] Wang Y. Yang H. Zi C. Wang Z. (2019). Transcriptomic analysis of the red and green light responses in Columba livia domestica. Biotech. 9:20. 10.1007/s13205-018-1551-1 30622858 PMC6314938

[B53] Webb R. Nicholas B. Gong J. G. Campbell B. K. Gutierrez C. G. Garverick H. A. (2003). Mechanisms regulating follicular development and selection of the dominant follicle. Reprod. Suppl. 61, 71–90. 14635928

[B54] Wells R. G. (1971). A comparison of red and white light and high and low dietary protein regimes for growing pullets. Brit. Poult. Sci. 12, 313–325. 10.1080/00071667108415887 5142745

[B55] Woodard A. E. Moore J. A. Wilson W. O. (1969). Effect of wave length of light on growth and reproduction in Japanese quail (Coturnix coturnix japonica). Poult. Sci. 48, 118–123 10.3382/ps.0480118 5355478

[B56] Woods D. Johnson A. L. (2005). Regulation of follicle-stimulating hormone-receptor messenger RNA in hen granulosa cells relative to follicle selection. Biol. Reprod. 72, 643–650. 10.1095/biolreprod.104.033902 15537865

[B57] Wucher V. Legeai F. Hedan B. Rizk G. Lagoutte L. Leeb T. (2017). FEELnc: a tool for long non-coding RNA annotation and its application to the dog transcriptome. Nucleic Acids Res. 45:57. 10.1093/nar/gkw1306 28053114 PMC5416892

[B58] Zhou P. Baumgarten S. C. Wu Y. Bennett J. Winston N. Hirshfeld-Cytron J.. (2013). IGF-I signaling is essential for FSH stimulation of AKT and steroidogenic genes in granulosa cells. Mol. Endocrinol. 27, 511–523. 10.1210/me.2012-1307 23340251 PMC3589673

[B59] Zhou S. Ma Y. Zhao D. Mi Y. Zhang C. (2020). Transcriptome profiling analysis of underlying regulation of growing follicle development in the chicken. Poult. Sci. 99, 2861–2872. 10.1016/j.psj.2019.12.067 32475419 PMC7597661

